# Supplementary material for: Experiences of general practice of children with complex and palliative care needs and their families: a qualitative study
Source: BMJ Open. 2021 Jan 6;11(1):e041476. doi: 10.1136/bmjopen-2020-041476 (PMC7789436; doi:10.1136/bmjopen-2020-041476)
Supplement: Author's manuscript [file bmjopen-2020-041476.draft_revisions.pdf]

BMJ Open is committed to open peer review. As part of this commitment we make the peer review history of every article we publish publicly available.

When an article is published we post the peer reviewers' comments and the authors' responses online. We also post the versions of the paper that were used during peer review. These are the versions that the peer review comments apply to.

The versions of the paper that follow are the versions that were submitted during the peer review process. They are not the versions of record or the final published versions. They should not be cited or distributed as the published version of this manuscript.

BMJ Open is an open access journal and the full, final, typeset and author-corrected version of record of the manuscript is available on our site with no access controls, subscription charges or pay-per-view fees (http://bmjopen.bmj.com).

If you have any questions on BMJ Open's open peer review process please email info.bmjopen@bmj.com

BMJ Open

Experiences of General Practice of children with complex and palliative care needs and their families: a qualitative study

Journal:	*BMJ Open*
Manuscript ID	bmjopen-2020-041476
Article Type:	Original research
Date Submitted by the Author:	09-Jun-2020
Complete List of Authors:	Mitchell, Sarah; The University of Sheffield<br>Harding, Stephanie; University of Warwick<br>Samani, Mohini; NIHR CRN West Midlands Young Persons Steering Group<br>Coad, Jane; University of Nottingham Faculty of Medicine and Health Sciences<br>Slowther, Anne-Marie; University of Warwick, Warwick Medical School<br>Dale, Jeremy; University of Warwick, Warwick Medical School
Keywords:	Community child health < PAEDIATRICS, Paediatric palliative care < PAEDIATRICS, PRIMARY CARE, PAEDIATRICS

SCHOLARONE™
Manuscripts

I, the Submitting Author has the right to grant and does grant on behalf of all authors of the Work (as defined in the below author licence), an exclusive licence and/or a non-exclusive licence for contributions from authors who are: i) UK Crown employees; ii) where BMJ has agreed a CC-BY licence shall apply, and/or iii) in accordance with the terms applicable for US Federal Government officers or employees acting as part of their official duties; on a worldwide, perpetual, irrevocable, royalty-free basis to BMJ Publishing Group Ltd ("BMJ") its licensees and where the relevant Journal is co-owned by BMJ to the co-owners of the Journal, to publish the Work in this journal and any other BMJ products and to exploit all rights, as set out in our licence.

The Submitting Author accepts and understands that any supply made under these terms is made by BMJ to the Submitting Author unless you are acting as an employee on behalf of your employer or a postgraduate student of an affiliated institution which is paying any applicable article publishing charge ("APC") for Open Access articles. Where the Submitting Author wishes to make the Work available on an Open Access basis (and intends to pay the relevant APC), the terms of reuse of such Open Access shall be governed by a Creative Commons licence – details of these licences and which Creative Commons licence will apply to this Work are set out in our licence referred to above.

Other than as permitted in any relevant BMJ Author's Self Archiving Policies, I confirm this Work has not been accepted for publication elsewhere, is not being considered for publication elsewhere and does not duplicate material already published. I confirm all authors consent to publication of this Work and authorise the granting of this licence.

For peer review only

Experiences of General Practice of children with complex and palliative care needs and their families: a qualitative study

Mitchell S, Harding S, Samani M, Slowther A-M, Coad J, Dale J.

Authors:

Dr Sarah Mitchell, MBChB FRCGP MSc PhD[1],
Miss Stephanie Harding[2],
Miss Mohini Samani[3],
Prof Anne-Marie Slowther MBChB MRCGP MA DPhil[2],
Prof Jane Coad RGN / RSCN PGDip BA BSc PhD[4],
Prof Jeremy Dale MA MBBS PhD FRCGP[2].

Corresponding Author:

Sarah Mitchell
Clinical Research Fellow, University of Sheffield
S.J.Mitchell@sheffield.ac.uk;
@MacGPSarah

Author affiliations:

1. University of Sheffield, Dept of Oncology and Metabolism, Weston Park Hospital, Whitham Road, Sheffield, S10 2SJ
2. Warwick Medical School, University of Warwick, Gibbet Hill Road, Coventry, CV4 7AL
3. NIHR CRN West Midlands Young Person's Steering Group
4. School of Health Sciences, Queens Medical Centre Campus, University of Nottingham, NG7 2HA

Word counts

Abstract: 249
Article: 2,864 (including quotes) (2,326 not including quotes)
Tables: 1
Boxes: 1

Authorship statement

SM, JD, A-MS and JC designed the study. SM conducted the interviews. SM and SH analysed the data, with input from MS as a representative of the PPI advisory group. JC, A-MS and JD revised the article critically for clarity and intellectual content. All authors have approved this version for submission.

For peer review only - http://bmjopen.bmj.com/site/about/guidelines.xhtml

Abstract

Background

The number of children and young people living with life-limiting and life-threatening conditions, who have long-term palliative care needs (physical, psychological, social and spiritual) is increasing rapidly. They receive healthcare from a range of specialist teams, but GPs and their teams tend to have little involvement.

Objectives

To investigate the views and experiences of general practice of children with life-limiting and life-threatening conditions, and their family members.

Study Design

Secondary thematic analysis of qualitative interview data.

Setting

West Midlands, UK.

Participants

14 families: 10 children aged 5-18 years with life-limiting and life-threatening conditions, and 21 of their family members.

Method

A total of 41 serial qualitative interviews were conducted. Secondary thematic analysis of all interview data relating to experiences of primary care.

Results

Three key themes emerged: (1) poor experiences of general practice cause children and families to feel isolated, (2) children and families value support from general practice, and (3) general practice has the potential to provide important aspects of care. Children and families reported benefits from fostering their relationship with their GP practice in order to access important aspects of care, including the assessment and management of acute illness, chronic disease and medication reviews, and holistic support.

Conclusion

Children with life-limiting and life-threatening conditions and their families value the involvement of general practice in the care, alongside their paediatric specialists. Ways of developing and providing such support as part of an integrated system of care need to be developed.

For peer review only - http://bmjopen.bmj.com/site/about/guidelines.xhtml

Keywords

Primary health care, General practice, Palliative care, Paediatrics, Child health

Strengths and limitations of this study

- The numbers of children with life-limiting and life-threatening conditions is rising, and there is evidence to suggest that more frequent contact with GPs, and continuity of care in general practice, is associated with decreased use of urgent and emergency care for these children.
- The role of GPs in the care of children with life-limiting and life-threatening conditions and their families, including aspects of palliative care, is poorly understood.
- This study provides insights into the support and care from general practice that children and their family members find most helpful from their perspective, including their strategies for accessing such care.

Funding statement

This study was funded by a National Institute for Health Research Doctoral Research Fellowship (DRF-2014-07-065).

Disclaimer

This article presents independent research funded in part by the National Institute for Health Research (NIHR). The views expressed are those of the authors and not necessarily those of the NHS, the NIHR or the Department of Health.

Competing interests

The authors have no competing interests

For peer review only - http://bmjopen.bmj.com/site/about/guidelines.xhtml

Introduction

The numbers of children and young people (hereafter referred to as "children") aged 0-19 years living with life-limiting conditions (for which there is no reasonable hope of cure) and life-threatening conditions (those for which curative treatment is feasible but may fail) is rising rapidly (1-3). As medical treatments and technology advance, these children are living with increasingly complex conditions. Their health is often fragile, with sudden and unanticipated deterioration leading to admission to hospital and intensive care. This has a huge impact on family life, and presents a number of challenges for healthcare services, including the management of complex clinical issues and the provision of adequate care and support, including palliative care, as close to home as possible (4).

General practitioners (GPs) and general practice teams have a role to play in the provision of healthcare to children with life-limiting and life-threatening conditions, who like all others, have primary care needs in addition to the specialist needs associated with their underlying conditions. For some children and their families, this includes aspects of palliative care (2). GPs can be key in terms of co-ordinating care from community and specialist teams, provision of prescriptions, support for family members and at transition into adult services (5). A recent epidemiological study has found that where children with life-limiting and life-threatening conditions attend appointments with their GP more frequently, and receive continuity of care with the same GP, they make less frequent use of urgent and emergency secondary care services (6). However, GPs have concerns about providing care to children with palliative care needs and their families including time-restraints, lack of specialist knowledge (particularly with rare conditions), and understanding of their role alongside paediatric colleagues (7, 8).

This study aimed to explore the role of general practice in the care of children with life-limiting and life-threatening conditions and their families, from their perspective, and consider how this might be developed to more effectively support the delivery of care to these children in the future.

Method

This study is a secondary analysis of data relating to primary care collected through a qualitative serial interview study with children and their family members, carried out in the West Midlands (detailed in Box 1 (9)). Children and family members participated in up to three interviews over 13 months from October 2016 to November 2017. The interviews were deliberately open and conversational to gain in-depth insights into the experiences and perceptions of healthcare. Communication skills, including responding to cues and the use of silence were employed during the

For peer review only - http://bmjopen.bmj.com/site/about/guidelines.xhtml

interviews to explore the views of children and family members in relation to palliative care where possible.

[Insert Box 1]

Recordings of interviews were transcribed verbatim, and anonymised transcripts uploaded into NVivo for data management. All data related to experiences of general practice were coded by SM, the dataset for this secondary analysis. Two researchers (SH and SM) independently coded this data using an inductive approach to thematic analysis as described by Braun & Clarke (10, 11). The emerging codes and concepts were discussed between the researchers weekly throughout the data analysis process, allowing for the development of the themes and decreasing lone researcher bias (12).

Patient and public involvement (PPI)

PPI was an integral part of the wider study and informed every stage of the research. A PPI team member supported this study, in particular providing feedback on the emerging themes and supporting dissemination.

Findings

Study population

A total of 14 families were recruited to the study, all registered with different GP practices, urban, inner-city and rural. Ten children (aged 5-18 years) and 21 family members took part in the interviews. The other four children were unable to take part either because they could not communicate verbally, or because they were too unwell. 41 serial interviews were conducted. In addition to specialist paediatric care, all the children received care from a local community children's nursing team, and six received care from a specialist paediatric palliative care team or children's hospice service. The study population is summarised in table 1.

[insert Table 1]

Qualitative findings

The children and their family members described a range of experiences of general practice both positive and negative. They did not tend to associate GPs with providing aspects of palliative care; all of the children and families who received palliative care services considered and conceptualised "palliative care" as distinct and separate from other services. Three overarching themes relating to general practice were identified:

Theme 1: Poor experiences of general practice cause children and families to feel isolated

For peer review only - http://bmjopen.bmj.com/site/about/guidelines.xhtml

For many, there was a lack of contact with GPs and practice teams. Several families had negative experiences and chose to avoid the GP surgery completely due to these experiences, and instead tended to contact their specialist teams for help. Most described their GPs as being of little or "*no help at all*" (M002). Many family members described a "*fight*" to access the support from obtain their GP practice that they required:

"I haven't got the power, the brain power, to deal with the doctors' surgery. Literally, it's just like I can't. The doctors' surgery is a no go. I avoid it. It will be my last call, if I have to." (M005)

Family members perceived the complexity of their child's condition, and their extensive specialist care, as potential barriers to GPs becoming more involved, with GPs lacking confidence around certain aspects of care, such as prescribing or treating acute medical problems.

"...an awful battle just to try and get antibiotics for her for a urine infection, it was terrible." (M003)

"As soon as [child]'s bowel problems started they just pulled back from everything. Even her basic [medications] and whatever she needed, it all just stopped." (M002)

Even with minor illness, families were advised to access hospital care, which they did not always agree was appropriate:

"...you're bringing this person up to the [children's hospital] who doesn't really need to be at the [children's hospital] and affecting everybody else because the GP won't come out." (M011)

On occasions, GPs declined to prescribe regular medications for the children. The rationale for this could be difficult for families to understand:

"Everything is like, oh, I can't prescribe this because it's unlicensed, or it's un-this, and simple things, [drug names for emollients], he wouldn't do it" (M005)

Other highly personal reasons caused a loss of trust in general practice. In one example, the GP was considered responsible for missing a significant diagnosis in the child:

"he didn't realise. You know, when you're talking life and death for your child, you can't make mistakes like that. But it was… and most GPs don't see things like this. It's because… they say it's rare, it doesn't feel rare to us in here [the hospital]" (F007).

In another, the GP was seen to have limited time to hear and understand a parent's stress:

"[the GP] gives me tablets all the time. What if the palpitations are something else? I avoid them now. They are so busy, over-stretched. I think the empathy has gone" (M013)

For peer review only - http://bmjopen.bmj.com/site/about/guidelines.xhtml

Theme 2: Children and families value support from general practice

Some families described positive experiences of primary care, and considered primary care as "*realistically the core of it all*" (M003), even when hospital-based, specialist teams mainly provided their care. Some had known a particular GP for many years, and highly valued this continuity, with the GP "*knowing our family*" (M006). For one family, it was the GP who *"finally realised"* (F004) that they were in need of extra support and arranged an onward referral to community children's nursing and palliative care services. Being able to rely on GPs to attend when children with complex needs had acute illness, so that they could avoid a trip to hospital, was important to families:

"They're good at coming out if he has a chest infection, they do come out, yeah. Because at the end of the day I've got a child that's on a sats monitor, and ventilator, and now a humidified circuit, and he's not well" (M014)

Children and their family members all expressed a desire for more support from the GP practice. They wished for a proactive offer of support, with recognition of their holistic care needs, through *"a general check on the normal everyday."(M005),* for example through a phone call. This was considered particularly important when there was a long time between hospital appointments. One child described this time as a time when "*no-one knows what is going on*" and specifically regarded the GP or a member of the practice team as someone who could "*check-in*" with him (C013).

"...that would be perfect if there was a somebody there that every six months just said, right, [name], how is everything going in the home, how is everything with .. all your meds, have you got any problems, is there anything I can do for you, can I phone anyone, do you need anything, just a someone." (M013).

Theme 3: Practical ways through which general practice could provide improved care.

Family members described the aspects of care that they wanted general practice teams to provide, and made efforts to "*consciously foster*" (M006) their relationships with GPs in order to access this care. Opportunities existed in chronic disease reviews, appointments for vaccinations and sick notes:

"I have always been quite proactive in taking my children to the GP not only when they're sick. I would take them [children] along for flu jabs and things like that, just so that they knew what they're like, so that when I take them when they're sick they know the difference" (M006)

Interventions that children and family members felt it would be helpful for general practice teams to offer included blood tests, so that they didn't have to travel long distances to hospital. Where repeat prescriptions were a particular cause of difficulty, members of the wider team, including practice

For peer review only - http://bmjopen.bmj.com/site/about/guidelines.xhtml

pharmacists had been instrumental in improving the situation through review of discharge summaries and updating a regular prescription:

"As soon as they get a letter from the hospital to say her meds changed they have someone ring me and we go through her meds." (M011)

Transition to adult services was a cause of concern for many families. They considered this a time when it would be helpful for support from general practice to "*really kick in*" (M003), particularly because organisation of healthcare services for adults differed significantly to those for children:

"I didn't realise that in adult care, a lot of help is from the GP and the community nurse, but we didn't know that because it's different at the [children's hospital]. , and when we went to the [hospital] this consultant, and neuro, said, they [primary care] have to [prescribe the medicines] but they won't do it" (M002)

The need for a consistent and familiar trusted contact within the surgery for children who may want to attend appointments alone, was highlighted:

"how the hell can I transition my son when the GP has never ever called … I'm trying to explain to [child], you will be old enough to go to the GP on your own. That's immense for a normal kid, but a kid with all [his conditions]… he won't talk about all of his…I want him to, because I think it would be a good idea if there was a particular person in the GP's" (M013)

Discussion

Summary

This study provides valuable insights into the highly variable experiences of general practice of children with life-limiting and life-threatening conditions and their family members. For some, there were some positive examples of general practice involvement in the child's care, but generally the relationship was experienced as unsatisfactory. This could result in avoidance, or years of battling to obtain the care and support they required. Both children and family members expressed a wish to receive more support from their GP practice, and importantly had developed strategies in order to access the support that they found most helpful.

Strengths & limitations

A major strength of this study is that it considered the experiences and perceptions of children and their families. The study population was relatively small, but it was diverse in terms of the children's condition and age. The participants were registered with 14 different practices, in a broad range of different settings. In-depth serial interviews, through which a relationship with children and their families could develop, led to rich, detailed data being elicited around many different aspects of

For peer review only - http://bmjopen.bmj.com/site/about/guidelines.xhtml

care. PPI was gained at several stages of the research, including the development of themes during the analysis to ensure the research remained representative and sensitive.

There is more research to be done to understand the experiences of younger children, young people at transition, and those with non-verbal communication. Further research into the role and response of primary care beyond general practice, including dentists, opticians and out of hours services for children with life-limiting and life-threatening conditions would also be valuable.

Comparison with existing literature

Care for children with complex and palliative care needs in general practice is an under-researched area of practice. The children and families who participated in this study described the particular aspects of care that would be helpful for primary care to provide, and their experiences of how such care could be delivered. The findings are in keeping with previous research, which has explored the experiences of GPs in palliative care of children with cancer (7, 8, 13), and the perspectives of bereaved parents (14). Continuity of GP care, effective communication to support care from both general practice and hospital-based, specialist care, and bereavement support, are areas described repeatedly as needing improvement. Furthermore, recent research suggests an association between more regular contact with GPs and less frequent use of urgent hospital care, which warrants further investigation (6). Transition from paediatric to adult services is also an area, described as an "overwhelming process", with a sense of loss of paediatric services, through which parents of children with complex care needs could benefit from continuity of care and support from their GP practice (15).

Implications for clinical practice and research

Recent research shows that the number of children with life-limiting or life-threatening conditions increased in England has risen from 32,975 in 2001/2002, to 86,625 in 2017/2018 (3). This is double the total GP headcount in England, which in March 2017 was 41,891 (33,921 whole time equivalent GPs) (16). The role and response of general practice in the healthcare of these children and their families, needs greater consideration.

The findings of this study suggest a need for a more systematic proactive approach, such as through prescribing and chronic disease reviews, to provide continuity of care and to build valuable therapeutic relationships with children with life-limiting and life-threatening conditions and their families. GPs and practice team members have the skills and ability to address the physical, psychological, social and spiritual aspects of care, through these relationships and as part of a wider multi-disciplinary team. They could play an increasingly important role in the identification of palliative care need and onward referral to specialist services, including communication with

For peer review only - http://bmjopen.bmj.com/site/about/guidelines.xhtml

specialists in paediatric palliative care and children's hospice services. Having a named GP within the practice who becomes the key point of contact for the family and the specialist team, becomes familiar with the needs of the child and family, and undertakes a regular review in which a care plan is agreed, would be of value.

Developing the skills, knowledge, expertise and confidence levels of GPs and other members of the practice team, perhaps those with a particular interest in paediatrics, or management of complexity, to assess acute illness in children with life-limiting and life-threatening conditions would also be of value. In theory, general practice is well placed to work with these children and families, however general practice is under-resourced and may not currently be in a position to prioritise this care.

At a healthcare system level, the need for collaborative partnership working between primary care clinicians and secondary care colleagues in order to improve the experiences of care of children with life-limiting and life-threatening conditions and their families is important. Interventions such as clear and personalised shared care plans, and shared electronic medical records, would be helpful. National professional bodies, including the Royal College of General Practitioners and the Royal College of Paediatrics and Child Health in the UK, have a key role to play in driving forward innovations and effective interventions to facilitate collaborative care. National and local commissioning strategies require focus on this population of children, and should support the delivery of their care in both hospital and community settings.

Conclusion

The findings from this study suggests that general practice should be playing a more important role in the provision of holistic, family-centred healthcare to children with life-limiting and life-threatening conditions and their family members. Children and families described multiple benefits to consciously fostering their relationship with general practice clinicians in order to access important aspects of care, including continuity. There are opportunities to engage with this through chronic disease and medication reviews. Clinicians in general practice could develop their skills and expertise in this area to provide assessment and management of acute illness, and long term holistic support, in close partnership with family members and paediatric specialists.

Additional information

Acknowledgements

The authors would like to acknowledge all of the children and families who took part in this study, and all of the Patient and Public Involvement representatives from Birmingham Children's Hospital, Acorns Children's Hospice and the NIHR CRN West Midlands Young Person's Steering Group who contributed to all aspects of the study.

For peer review only - http://bmjopen.bmj.com/site/about/guidelines.xhtml

Ethical approval

Ethical approval was granted by the UK Health Research Authority on 14th September 2016 (IRAS ID: 196816, REC reference: 16/WM/0272, Sponsor: University of Warwick). The data was anonymised and personal identifiers removed to protect confidentiality, as described in the methods.

References

1. Fraser LK, Miller M, Hain R, et al. Rising national prevalence of life-limiting conditions in children in England. Pediatr. 2012;129(4):e923-9.
2. Together for Short Lives. Children's Palliative Care Definitions. https://www.togetherforshortlives.org.uk/changing-lives/supporting-care-professionals/introduction-childrens-palliative-care/categories-of-life-limiting-conditions/.
3. Fraser LK. Gibson-Smith D, Jarvis S. et al. Make Every Child Count. Estimating current and future prevalence of children and young people with life-limiting conditions in the United Kingdom. York: University of York; 2020.
4. Collins A, Hennessy-Anderson N, Hosking S, et al. Lived experiences of parents caring for a child with a life-limiting condition in Australia: A qualitative study. Palliat Med. 2016;30(10):950-9.
5. van der Geest IM, Bindels P, Pluijm S, et al. Palliative care in children with cancer: implications for general practice. Br J Gen Pract. 2016;66(653):599-600.
6. Jarvis SW, Parslow R, Hewitt CE, et al. GPs' role in caring for children and young people with life limiting conditions – a retrospective cohort study. Br J Gen Pract. 2020;70(693): e221-e229. DOI: https://doi.org/10.3399/bjgp20X7082332020:1-9.
7. Neilson S, Gibson F, Jeffares S, Greenfield SM. GPs and paediatric oncology palliative care: a Q methodological study. BMJ Support Palliat Care. 2017; bmjspcare-2015-000852. doi: 10.1136/bmjspcare-2015-000852.2017.
8. Neilson SJ, Gibson F, Greenfield SM. Pediatric Oncology Palliative Care: Experiences of General Practitioners and Bereaved Parents. J Palliat Care Med. 2015;5(2):214.
9. Mitchell S, Slowther A-M, Coad J, Dale J. The journey through care: study protocol for a longitudinal qualitative interview study to investigate the healthcare experiences and preferences of children and young people with life-limiting and life-threatening conditions and their families in the West Midlands, UK. BMJ Open. 2018;8(1).
10. Braun V, Clarke V. Using thematic analysis in psychology. Qualitative Research in Psychology. 2006;3(2):77-101.
11. Braun V, Clarke V. Successful Qualitative Research. London: SAGE; 2013.
12. Burnard P, Gill P, Stewart K, Treasure E, Chadwick B. Analysing and presenting qualitative data. Br Dent J. 2008;204(8):429-32.
13. van der Geest IM, Bindels PJ, Pluijm SM, et al. Home-Based Palliative Care for Children With Incurable Cancer: Long-term Perspectives of and Impact on General Practitioners. J Pain Sympt Manage. 2017;53(3):578-87.
14. van der Geest IM, Darlington AS, Streng IC, et al. Parents' experiences of pediatric palliative care and the impact on long-term parental grief. J Pain Symptom Manage. 2014;47(6):1043-53.
15. Brown M, Higgins A, MacArthur J. Transition from child to adult health services: A qualitative study of the views and experiences of families of young adults with intellectual disabilities. J Clin Nurs. 2020;29(1-2):195-207.
16. NHS Digital. General and Personal Medical Services, England As at 31 March 2017, Experimental statistics https://digital.nhs.uk/data-and-information/publications/statistical/general-practice-workforce-archive/as-at-31-march-2017-experimental-statistics2018

For peer review only - http://bmjopen.bmj.com/site/about/guidelines.xhtml

Box 1: Summary of main study

Aim of the study

To examine the delivery of healthcare, including palliative care, for children with life-limiting and life-threatening conditions and their families. The study uses a realist approach to provide understanding into how palliative care is delivered most effectively, and when, leading to policy relevant recommendations.

Methods:

1. 41 in-depth serial interviews with 31 participants from 14 families; 10 children with life-limiting or life-threatening conditions and 21 family members. A broad topic guide covered experiences of all aspects of healthcare, including palliative care where possible.
2. Four focus groups with children's palliative care professionals.
3. Thematic analysis, followed by application of a realist logic, leading to a programme theory for the delivery of palliative care.

Findings:

Children and their families are vulnerable experts, negotiating a healthcare system that can be rigid and fragmented. The delivery of palliative care depends on interpersonal relationships with healthcare professionals, who are able to bear witness to the child and family situation, underpinned by trust and respect. Important child and family outcomes include feeling heard and supported. In organisations, an environment that values these relationships and legitimises palliative care as an approach through leadership and role modelling as well as the development of specialist services, is important.

Conclusion:

The study provides increased understanding and description of important child and family outcomes that underpin policy goals in palliative care, achieved in certain contexts. Future service and commissioning models should propose a whole system approach, with greater emphasis on the need for trusted relationships, nurturing and supporting professionals who have the motivation and capacity to provide palliative care to children, and should integrate specialist

For peer review only - http://bmjopen.bmj.com/site/about/guidelines.xhtml

Table 1: Summary of study population.

Family	Participants and identifier	Child's age at recruitment	Type of condition	Male or Female	GP practice area	Palliative care service involved	Number of interviews
1	Child (C001)<br>Mother (M001)<br>Father (F001)	5	Cancer	M	Urban	No	3
2	Child (C002)<br>Mother (M002)<br>Brother (B002)	17	Congenital	F	Inner-city	Yes	3
3	Mother (M003)<br>Father (F003)	8	Congenital	F	Inner-city	Yes	3
4	Father (F004)	8	Congenital	F	Inner-city	Yes	1
5	Child (C005)<br>Mother (M005)	6	Congenital	M	Urban	No	3
6	Mother (M006)	18	Congenital	M	Inner-city	No	3
7	Child (C007)<br>Mother (M007)<br>Father (F007)	7	Cancer	M	Urban	No	3
8	Child (C008)<br>Mother (M008)<br>Brother (B008)	5	Congenital	M	Inner-city	No	3
9	Child (C009)<br>Mother (M009)<br>Father (F009)	11	Cancer	F	Inner-city	No	3
10	Mother (M010)	5	Congenital	M	Urban	Yes	1
11	Child (C011)<br>Mother (M011)<br>Step-father (F011)	17	Congenital	F	Urban	No	3
12	Child (C012)<br>Mother (M012)	14	Cancer	M	Urban	No	1
13	Child (C013)<br>Mother (M013)	14	Cancer	M	Urban	Yes	2
14	Child (C014)<br>Mother (M014)	10	Congenital	M	Rural	Yes	3

For peer review only - http://bmjopen.bmj.com/site/about/guidelines.xhtml

For peer review only - http://bmjopen.bmj.com/site/about/guidelines.xhtml

COREQ (COnsolidated criteria for REporting Qualitative research) Checklist

A checklist of items that should be included in reports of qualitative research. You must report the page number in your manuscript where you consider each of the items listed in this checklist. If you have not included this information, either revise your manuscript accordingly before submitting or note N/A.

Topic	Item No.	Guide Questions/Description	Reported on Page No.
**Domain 1: Research team and reflexivity**			
Personal characteristics			
Interviewer/facilitator	1	Which author/s conducted the interview or focus group?	
Credentials	2	What were the researcher's credentials? E.g. PhD, MD	
Occupation	3	What was their occupation at the time of the study?	
Gender	4	Was the researcher male or female?	
Experience and training	5	What experience or training did the researcher have?	
Relationship with participants			
Relationship established	6	Was a relationship established prior to study commencement?	
Participant knowledge of the interviewer	7	What did the participants know about the researcher? e.g. personal goals, reasons for doing the research	
Interviewer characteristics	8	What characteristics were reported about the inter viewer/facilitator? e.g. Bias, assumptions, reasons and interests in the research topic	
**Domain 2: Study design**			
Theoretical framework			
Methodological orientation and Theory	9	What methodological orientation was stated to underpin the study? e.g. grounded theory, discourse analysis, ethnography, phenomenology, content analysis	
Participant selection			
Sampling	10	How were participants selected? e.g. purposive, convenience, consecutive, snowball	
Method of approach	11	How were participants approached? e.g. face-to-face, telephone, mail, email	
Sample size	12	How many participants were in the study?	
Non-participation	13	How many people refused to participate or dropped out? Reasons?	
Setting			
Setting of data collection	14	Where was the data collected? e.g. home, clinic, workplace	
Presence of non-participants	15	Was anyone else present besides the participants and researchers?	
Description of sample	16	What are the important characteristics of the sample? e.g. demographic data, date	
Data collection			
Interview guide	17	Were questions, prompts, guides provided by the authors? Was it pilot tested?	
Repeat interviews	18	Were repeat inter views carried out? If yes, how many?	
Audio/visual recording	19	Did the research use audio or visual recording to collect the data?	
Field notes	20	Were field notes made during and/or after the inter view or focus group?	
Duration	21	What was the duration of the inter views or focus group?	
Data saturation	22	Was data saturation discussed?	
Transcripts returned	23	Were transcripts returned to participants for comment and/or	

For peer review only

Topic	Item No.	Guide Questions/Description	Reported on Page No.
		correction?	
**Domain 3: analysis and findings**			
Data analysis			
Number of data coders	24	How many data coders coded the data?	
Description of the coding tree	25	Did authors provide a description of the coding tree?	
Derivation of themes	26	Were themes identified in advance or derived from the data?	
Software	27	What software, if applicable, was used to manage the data?	
Participant checking	28	Did participants provide feedback on the findings?	
Reporting			
Quotations presented	29	Were participant quotations presented to illustrate the themes/findings? Was each quotation identified? e.g. participant number	
Data and findings consistent	30	Was there consistency between the data presented and the findings?	
Clarity of major themes	31	Were major themes clearly presented in the findings?	
Clarity of minor themes	32	Is there a description of diverse cases or discussion of minor themes?	

Developed from: Tong A, Sainsbury P, Craig J. Consolidated criteria for reporting qualitative research (COREQ): a 32-item checklist for interviews and focus groups. *International Journal for Quality in Health Care*. 2007. Volume 19, Number 6: pp. 349 – 357

Once you have completed this checklist, please save a copy and upload it as part of your submission. DO NOT include this checklist as part of the main manuscript document. It must be uploaded as a separate file.

For peer review only

Experiences of General Practice of children with complex and palliative care needs and their families: a qualitative study

Journal:	*BMJ Open*
Manuscript ID	bmjopen-2020-041476.R1
Article Type:	Original research
Date Submitted by the Author:	14-Oct-2020
Complete List of Authors:	Mitchell, Sarah; The University of Sheffield; University of Warwick Warwick Medical School, Academic Unit of Primary Care<br>Harding, Stephanie; University of Warwick<br>Samani, Mohini; NIHR CRN West Midlands Young Persons Steering Group<br>Slowther, Anne-Marie; University of Warwick, Warwick Medical School<br>Coad, Jane; University of Nottingham Faculty of Medicine and Health Sciences<br>Dale, Jeremy; University of Warwick, Warwick Medical School
**Primary Subject Heading**:	General practice / Family practice
Secondary Subject Heading:	Palliative care, Paediatrics
Keywords:	Community child health < PAEDIATRICS, Paediatric palliative care < PAEDIATRICS, PRIMARY CARE, PAEDIATRICS

For peer review only - http://bmjopen.bmj.com/site/about/guidelines.xhtml

I, the Submitting Author has the right to grant and does grant on behalf of all authors of the Work (as defined in the below author licence), an exclusive licence and/or a non-exclusive licence for contributions from authors who are: i) UK Crown employees; ii) where BMJ has agreed a CC-BY licence shall apply, and/or iii) in accordance with the terms applicable for US Federal Government officers or employees acting as part of their official duties; on a worldwide, perpetual, irrevocable, royalty-free basis to BMJ Publishing Group Ltd ("BMJ") its licensees and where the relevant Journal is co-owned by BMJ to the co-owners of the Journal, to publish the Work in this journal and any other BMJ products and to exploit all rights, as set out in our licence.

The Submitting Author accepts and understands that any supply made under these terms is made by BMJ to the Submitting Author unless you are acting as an employee on behalf of your employer or a postgraduate student of an affiliated institution which is paying any applicable article publishing charge ("APC") for Open Access articles. Where the Submitting Author wishes to make the Work available on an Open Access basis (and intends to pay the relevant APC), the terms of reuse of such Open Access shall be governed by a Creative Commons licence – details of these licences and which Creative Commons licence will apply to this Work are set out in our licence referred to above.

Other than as permitted in any relevant BMJ Author's Self Archiving Policies, I confirm this Work has not been accepted for publication elsewhere, is not being considered for publication elsewhere and does not duplicate material already published. I confirm all authors consent to publication of this Work and authorise the granting of this licence.

For peer review only

Experiences of General Practice of children with complex and palliative care needs and their families: a qualitative study

Mitchell S, Harding S, Samani M, Slowther A-M, Coad J, Dale J.

Authors:

Dr Sarah Mitchell, MBChB FRCGP MSc PhD[1],
Miss Stephanie Harding[2],
Miss Mohini Samani[3],
Prof Anne-Marie Slowther MBChB MRCGP MA DPhil[2],
Prof Jane Coad RGN / RSCN PGDip BA BSc PhD[4],
Prof Jeremy Dale MA MBBS PhD FRCGP[2].

Corresponding Author:

Sarah Mitchell
Clinical Research Fellow, University of Sheffield
S.J.Mitchell@sheffield.ac.uk;
@MacGPSarah

Author affiliations:

1. University of Sheffield, Dept of Oncology and Metabolism, Weston Park Hospital, Whitham Road, Sheffield, S10 2SJ
2. Warwick Medical School, University of Warwick, Gibbet Hill Road, Coventry, CV4 7AL
3. NIHR CRN West Midlands Young Person's Steering Group
4. School of Health Sciences, Queens Medical Centre Campus, University of Nottingham, NG7 2HA

Word counts

Abstract: 247
Article: 3,496 (including quotes) (2,957 not including quotes)
Tables: 1

Authorship statement

SM, JD, A-MS and JC designed the study. SM conducted the interviews. SM and SH analysed the data, with input from MS as a representative of the PPI advisory group. JC, A-MS and JD revised the article critically for clarity and intellectual content. All authors have approved this version for submission.

For peer review only - http://bmjopen.bmj.com/site/about/guidelines.xhtml

Abstract

Background

The number of children and young people living with life-limiting and life-threatening conditions, who have long-term palliative care needs (physical, psychological, social and spiritual) is increasing rapidly. They receive healthcare from a range of specialist teams, but GPs and their teams tend to have little involvement.

Aim

To investigate the views and experiences of general practice of children with life-limiting and life-threatening conditions, and their family members.

Study Design and Setting

Secondary thematic analysis of qualitative interview data from a study carried out in the West Midlands, UK.

Method

41 serial interviews with 31 participants from 14 families: 10 children aged 5-18 years with life-limiting and life-threatening conditions, and 21 of their family members. Secondary thematic analysis of all interview data relating to experiences of primary care.

Results

Three key themes emerged: (1) poor experiences of general practice cause children and families to feel isolated, (2) children and families value support from general practice, and (3) general practice has the potential to provide important aspects of care. Children and families reported benefits from fostering their relationship with their general practice in order to access important aspects of care, including the assessment and management of acute illness, chronic disease and medication reviews, and holistic support.

Conclusion

Children with life-limiting and life-threatening conditions and their families value the involvement of general practice in the care, alongside their paediatric specialists. Ways of developing and providing such support as part of an integrated system of care need to be developed.

Keywords

Primary health care, General practice, Palliative care, Paediatrics, Child health

For peer review only - http://bmjopen.bmj.com/site/about/guidelines.xhtml

How this fits in

The numbers of children with life-limiting and life-threatening conditions is rising. More frequent contact with GPs, and continuity of care in general practice, is associated with decreased use of urgent and emergency care for these children. However, the role of general practice in the care of children with life-limiting and life-threatening conditions and their families, including aspects of palliative care, is poorly understood. This study provides insights into the support and care from general practice that children and their family members find most helpful from their perspective, including their strategies for accessing such care.

For peer review only

Introduction

The numbers of children and young people (hereafter referred to as "children") aged 0-19 years living with life-limiting conditions (for which there is no reasonable hope of cure) and life-threatening conditions (those for which curative treatment is feasible but may fail) is rising rapidly (1-3). As medical treatments and technology advance, these children are living with increasingly complex conditions. Their health is often fragile, with sudden and unanticipated deterioration leading to admission to hospital and intensive care. This has a huge impact on family life, and presents a number of challenges for healthcare services, including the management of complex clinical issues and the provision of adequate care and support, including palliative care, as close to home as possible (4).

The involvement of general practitioners (GPs) and primary care teams in the provision of healthcare to children with life-limiting and life-threatening conditions can be variable. These children, like all others, have primary care needs in addition to the specialist needs associated with their underlying conditions. Those with life-limiting and life-threatening conditions may also have associated palliative care needs (2). GPs have a key role in the delivery of "core" palliative care to all patients who need it. For children, this would be alongside community nursing and specialist paediatric palliative care colleagues. However, GPs have expressed significant concerns about this role in palliative care for children, including a lack of time to be involved, lack of specialist knowledge (particularly with rare conditions), and understanding of their role alongside paediatric colleagues (5, 6). However, GPs have the potential to provide many vital aspects of palliative care, including the co-ordination of care from community and specialist teams, provision of prescriptions, holistic support for family members (including during bereavement) and support when children transition from paediatric to adult palliative care services (7). Furthermore, frequent appointments with a GP and continuity of GP care for children with life-limiting and life-threatening conditions is associated with less frequent use of urgent and emergency secondary care services (8).

This study aimed to explore the role of general practice in the care of children with life-limiting and life-threatening conditions and their families, from their perspective, and consider how this might be developed to more effectively support the delivery of care to these children in the future.

Method

This study is a secondary analysis of data relating to primary care collected during a qualitative serial interview study carried out from October 2016 to November 2017 in the West Midlands, UK (9). The overall aim of the study was to examine the delivery of healthcare, including palliative care, for children with life-limiting and life-threatening conditions and their families, using a realist approach

For peer review only - http://bmjopen.bmj.com/site/about/guidelines.xhtml

to provide understanding into how palliative care is delivered most effectively, and when, leading to policy relevant recommendations. Longitudinal interviews provided further benefits, including opportunities for rapport building and observation of changing needs and experiences of healthcare over time (10).

Children and family members were provided with information about the study by their clinical teams, or through information (leaflets and posters) that were in public areas including waiting rooms of hospital outpatient departments, and directly approach the research team. Each child and family member participated in up to three interviews over the 13-month period, either individually or as a family group. Qualitative research methods were most appropriate in order to elicit and explore the views and perceptions of children and their families. The interview process, including whether children wished to be interviewed alone or with their family members on each occasion, the timing and intervals between interviews, were individually considered according to the needs of each child and family. The interviews were open and conversational, and advanced communication skills, including responding to cues and the use of silence were employed. This allowed exploration of potentially sensitive issues, including experiences of palliative care as both a broad approach to care, and when the child and family had contact with specialist paediatric palliative care services.

Consent procedures were designed with the aim of obtaining written and / or verbal consent and agreement from every individual for every interview. For children under the age of 16, written consent was obtained from the parent and then verbal or written agreement obtained from the child. An agreement to participate form was completed by the child if they chose to do so, in order to respect their autonomy in so far as was possible.

Recordings of interviews were transcribed verbatim, and anonymised transcripts uploaded into NVivo for data management. All data related to experiences of general practice were coded by SM and formed the dataset for this secondary analysis. Two researchers (SH and SM) independently coded the data using an inductive approach to thematic analysis as described by Braun & Clarke (11, 12). The emerging codes and concepts were discussed between the researchers weekly throughout the data analysis process, allowing for the development of the themes and decreasing lone researcher bias (13).

Patient and public involvement (PPI)

PPI was an integral part of the wider study and informed every stage of the research. A group of PPI representatives was recruited from three existing advisory groups (based at Birmingham Children's Hospital, Acorns Children's Hospice and the NIHR Clinical Research Network in the West Midlands).

For peer review only - http://bmjopen.bmj.com/site/about/guidelines.xhtml

The PPI representatives were aged between nine and 25 years, and included young people with life-limiting conditions and siblings. The group were consulted at all stages of the research, from the study design and objectives to the format of interviews for children, and dissemination activities. A PPI representative (MS) volunteered to support this sub-analysis, providing feedback on the emerging themes and supporting dissemination.

Findings

Study sample

A total of 14 families were recruited to the study, all registered with different GP practices, urban, inner-city and rural. Ten children (aged 5-18 years) and 21 family members took part in the interviews. Children took part in the interviews either with their family members or alone, according to their preference on the day of the interview. Four children were unable to take part either because they could not communicate verbally, or because they were too unwell. 41 interviews were conducted in total. All of the children received care from a local community children's nursing team, and six received care from a specialist paediatric palliative care team or children's hospice service. The study population is summarised in table 1.

[insert Table 1]

Qualitative findings

The children and their family members described a range of experiences of general practice both positive and negative. They did not tend to associate GPs with providing aspects of palliative care; all of the children and families who received palliative care services considered and conceptualised "palliative care" as distinct and separate from other services. Three overarching themes relating to general practice were identified:

Theme 1: Poor experiences of general practice cause children and families to feel isolated

For many, there was a lack of contact with GPs and practice teams. Several families had negative experiences and chose to avoid the GP surgery completely due to these experiences, tending to contact their specialist teams for help instead. Most described their GPs as being of little or "*no help at all*" (M002). Many family members described a "*fight*" to access the support from obtain their GP practice that they required:

"I haven't got the power, the brain power, to deal with the doctors' surgery. Literally, it's just like I can't. The doctors' surgery is a no go. I avoid it. It will be my last call, if I have to." (M005)

For peer review only - http://bmjopen.bmj.com/site/about/guidelines.xhtml

Negative experiences led to a loss of trust in the GP that family members felt unable to reconcile. In these circumstances, there was a sense of unfortunate loss of an important source of support. In one example, the GP was considered responsible for missing a significant diagnosis (cancer) in the child:

"he didn't realise. You know, when you're talking life and death for your child, you can't make mistakes like that. But it was… and most GPs don't see things like this. It's because… they say it's rare, it doesn't feel rare to us in here [the hospital]" (F007).

Some family members had tried to access support from their GP for their own distress related to their child's condition. They sought a listening ear and validation of their distress, but perceived that the GP had limited time to hear and understand, and would focus on providing a medical solution such as an antidepressant tablet. This led to further frustration:

"[the GP] gives me tablets all the time. What if the palpitations are something else? I avoid them now. They are so busy, over-stretched. I think the empathy has gone" (M013)

Another frustration arose on occasions where GPs declined to prescribe regular medications for the children. Family members, who were experts in the management of their children's conditions, found it difficult to understand the rationale for this:

"Everything is like, oh, I can't prescribe this because it's unlicensed, or it's un-this, and simple things, [drug names for emollients], he wouldn't do it" (M005)

Family members recognised that the complexity of their child's condition, and their extensive specialist care, as potential barriers to GPs becoming more involved, with GPs lacking confidence around certain aspects of care, such as prescribing or treating acute medical problems.

"...an awful battle just to try and get antibiotics for her for a urine infection, it was terrible." (M003)

"As soon as [child]'s [life-limiting condition] problems started they just pulled back from everything. Even her basic [medications] and whatever she needed, it all just stopped." (M002)

Even with minor illness, families were advised to access hospital care, which they did not always agree was appropriate:

"...you're bringing this person up to the [children's hospital] who doesn't really need to be at the [children's hospital] and affecting everybody else because the GP won't come out." (M011)

Theme 2: Children and families value support from general practice

Some families described positive experiences of general practice, considering their GP to be "*realistically the core of it all*" (M003), even when hospital-based, specialist teams mainly provided

For peer review only - http://bmjopen.bmj.com/site/about/guidelines.xhtml

their care. Some had known a particular GP for many years, and highly valued this continuity, with the GP "*knowing our family*" (M006). For one family, it was the GP who *"finally realised"* (F004) that they were in need of extra support and arranged an onward referral to community children's nursing and palliative care services. Being able to consider the GP as an important member of their healthcare team, through, for example, their attendance when the child had an acute illness, so that they could avoid a trip to hospital, made a significant difference to families where this was the case:

"They're good at coming out if he has a chest infection, they do come out, yeah. Because at the end of the day I've got a child that's on a sats monitor, and ventilator, and now a humidified circuit, and he's not well" (M014)

Children and their family members all expressed a desire for more support from the GP practice. They wished for a proactive offer of support, with recognition of their holistic care needs, through *"a general check on the normal everyday."(M005),* for example through a phone call. This was considered particularly important when there was a long time between hospital appointments. One child described this time as a time when "*no-one knows what is going on*" and specifically regarded the GP or a member of the practice team as someone who could "*check-in*" with him (C013).

"...that would be perfect if there was a somebody there that every six months just said, right, [name], how is everything going in the home, how is everything with .. all your meds, have you got any problems, is there anything I can do for you, can I phone anyone, do you need anything, just a someone." (M013).

Theme 3: Practical ways through which general practice could provide improved care.

Family members described the aspects of care that they wanted general practice teams to provide, and made efforts to "*consciously foster*" (M006) their relationships with GPs in order to access this care. Opportunities existed in chronic disease reviews, appointments for vaccinations and sick notes:

"I have always been quite proactive in taking my children to the GP not only when they're sick. I would take them [children] along for flu jabs and things like that, just so that they knew what they're like, so that when I take them when they're sick they know the difference" (M006)

Interventions that children and family members felt it would be helpful for general practice teams to offer included blood tests, so that they didn't have to travel long distances to hospital. Where repeat prescriptions were a particular cause of difficulty, members of the wider team, including practice pharmacists had been instrumental in improving the situation through review of discharge summaries and updating a regular prescription:

For peer review only - http://bmjopen.bmj.com/site/about/guidelines.xhtml

"As soon as they get a letter from the hospital to say her meds changed they have someone ring me and we go through her meds." (M011)

Transition to adult services was a cause of concern for many families. They considered this a time when it would be helpful for support from general practice to "*really kick in*" (M003), particularly because organisation of healthcare services for adults differed significantly to those for children:

"I didn't realise that in adult care, a lot of help is from the GP and the community nurse, but we didn't know that because it's different at the [children's hospital]. , and when we went to the [hospital] this consultant, and neuro, said, they [primary care] have to [prescribe the medicines] but they won't do it" (M002)

The need for a consistent and familiar trusted contact within the surgery for children who may want to attend appointments alone, was highlighted:

"how the hell can I transition my son when the GP has never ever called … I'm trying to explain to [child], you will be old enough to go to the GP on your own. That's immense for a normal kid, but a kid with all [his conditions]… he won't talk about all of his…I want him to, because I think it would be a good idea if there was a particular person in the GP's" (M013)

Discussion

Summary

This study provides valuable insights into the highly variable experiences of general practice of children with life-limiting and life-threatening conditions and their family members. For some, there were some positive examples of general practice involvement in the child's care. Generally, the relationship was experienced as unsatisfactory. This could result in a loss of trust, avoidance, or years of battling to obtain the care and support required. Both children and family members expressed a wish to receive more support from their GP practice, and importantly had developed strategies in order to access the support that they found most helpful.

Strengths & limitations

A strength of this study is that it considered the experiences and perceptions general practice from the perspective of children and their families. Family members tended to talk more about experiences of general practice, which is likely to reflect their key involvement in liaising and negotiating services from GPs. This was a subanalysis of a wider study that did not specifically focus on experiences of general practice, and as such, the dataset is limited. The specific views of children are worthy of further exploration.

For peer review only - http://bmjopen.bmj.com/site/about/guidelines.xhtml

The study population was relatively small, but it was diverse in terms of the children's condition and age. The participants were registered with 14 different practices, in a broad range of different settings. In-depth serial interviews, through which a relationship with children and their families could develop, led to rich, detailed data elicited around many different aspects of care. PPI was gained at several stages of the research, including the development of themes during the analysis to ensure the research remained representative and sensitive.

There is more research to be done to understand the experiences of younger children, young people at transition, and those with non-verbal communication. Further research into the role and response of primary care beyond general practice, including dentists, opticians and out of hours services for children with life-limiting and life-threatening conditions would also be valuable.

Comparison with existing literature

Care for children with complex and palliative care needs in general practice is an under-researched area of practice. The children and families who participated in this study described the particular aspects of care that would be helpful for primary care to provide, and their experiences of how such care could be delivered. The findings are in keeping with the current, limited evidence base, which has explored the experiences of GPs in palliative care of children with cancer (7, 8, 14), and the perspectives of bereaved parents (15). Continuity of care in general practice, effective communication with hospital-based, specialist care, and bereavement support, are areas described repeatedly as needing improvement. Furthermore, the association between more regular contact with GPs and less frequent use of urgent hospital care warrants further investigation in order to understand the factors that result in this outcome for some children, and the details of their relationships with general practice (6). A particularly important area for consideration is transition from paediatric to adult services. This is described as an "overwhelming process" by some parents, with a sense of loss of paediatric services. Continuity of care and support from the GP practice in this scenario has many potential benefits for both parents and children with complex and palliative care needs but is often overlooked (16).

Implications for clinical practice and research

Recent research shows that the number of children with life-limiting or life-threatening conditions increased in England has risen from 32,975 in 2001/2002, to 86,625 in 2017/2018 (3). This is double the total GP headcount in England, which in March 2017 was 41,891 (33,921 whole time equivalent GPs) (17). The role and response of general practice in the healthcare of these children and their families, needs greater consideration.

For peer review only - http://bmjopen.bmj.com/site/about/guidelines.xhtml

The findings of this study suggest a need for a more systematic proactive approach, such as through prescribing and chronic disease reviews, to provide continuity of care and to build valuable therapeutic relationships with children with life-limiting and life-threatening conditions and their families. GPs and practice team members have the skills and ability to address the physical, psychological, social and spiritual aspects of care, through these relationships and as part of a wider multi-disciplinary team. These aspects of care may not necessarily be associated with "palliative" care, but they provide an important foundation and are integral to the delivery of palliative care to children, which by the nature of children's conditions, may be needed over a prolonged time period. GPs could play an increasingly important role in the identification of palliative care need and onward referral to specialist services, including communication with specialists in paediatric palliative care and children's hospice services. Having a named GP within the practice who becomes the key point of contact for the family and the specialist team, becomes familiar with the needs of the child and family, and undertakes a regular review in which a care plan is agreed, would be of value.

Developing the skills, knowledge, expertise and confidence levels of GPs and other members of the practice team, perhaps those with a particular interest in paediatrics, or management of complexity, to assess acute illness in children with life-limiting and life-threatening conditions would also be of value. In theory, general practice is well placed to work with these children and families, however general practice is under-resourced and may not currently be in a position to prioritise this care.

At a healthcare system level, the need for collaborative partnership working between primary care clinicians and secondary care colleagues in order to improve the experiences of care of children with life-limiting and life-threatening conditions and their families is important. Interventions such as clear and personalised shared care plans, and shared electronic medical records, would be helpful. National professional bodies, including the Royal College of General Practitioners and the Royal College of Paediatrics and Child Health in the UK, have a key role to play in driving forward innovations and effective interventions to facilitate collaborative care. National and local commissioning strategies require focus on this population of children, and should support the delivery of their care in both hospital and community settings.

Conclusion

The findings from this study suggests that general practice should be playing a more important role in the provision of holistic, family-centred healthcare to children with life-limiting and life-threatening conditions and their family members. Children and families described multiple benefits to consciously fostering their relationship with general practice clinicians in order to access important aspects of care, including continuity. There are opportunities to engage with this through

For peer review only - http://bmjopen.bmj.com/site/about/guidelines.xhtml

chronic disease and medication reviews. Clinicians in general practice could develop their skills and expertise in this area to provide assessment and management of acute illness, and long term holistic support, in close partnership with family members and paediatric specialists.

Additional information

Funding statement

This study was funded by a National Institute for Health Research Doctoral Research Fellowship (DRF-2014-07-065).

Disclaimer

This article presents independent research funded in part by the National Institute for Health Research (NIHR). The views expressed are those of the authors and not necessarily those of the NHS, the NIHR or the Department of Health.

Competing interests

The authors have no competing interests

Acknowledgements

The authors would like to acknowledge all of the children and families who took part in this study, and all of the Patient and Public Involvement representatives from Birmingham Children's Hospital, Acorns Children's Hospice and the NIHR CRN West Midlands Young Person's Steering Group who contributed to all aspects of the study.

Ethical approval

Ethical approval was granted by the UK Health Research Authority on 14th September 2016 (IRAS ID: 196816, REC reference: 16/WM/0272, Sponsor: University of Warwick). The data was anonymised and personal identifiers removed to protect confidentiality, as described in the methods.

Data sharing

Individual deidentified participant data that underlie the results published in this article will be made available from 3 months until 3 years following the article publication to investigators with a methodologically sound proposal and whose proposed use of the data has been approved by an independent review committee identified for this purpose, to achieve the aims in the approved proposal. Proposals can be submitted to the corresponding author.

For peer review only - http://bmjopen.bmj.com/site/about/guidelines.xhtml

References

1. Fraser LK, Miller M, Hain R, et al. Rising national prevalence of life-limiting conditions in children in England. Pediatr. 2012;129(4):e923-9.
2. Together for Short Lives. Children's Palliative Care Definitions. https://www.togetherforshortlives.org.uk/changing-lives/supporting-care-professionals/introduction-childrens-palliative-care/categories-of-life-limiting-conditions/.
3. Fraser LK. Gibson-Smith D, Jarvis S. et al. Make Every Child Count. Estimating current and future prevalence of children and young people with life-limiting conditions in the United Kingdom. York: University of York; 2020.
4. Collins A, Hennessy-Anderson N, Hosking S, et al. Lived experiences of parents caring for a child with a life-limiting condition in Australia: A qualitative study. Palliat Med. 2016;30(10):950-9.
5. Neilson S, Gibson F, Jeffares S, Greenfield SM. GPs and paediatric oncology palliative care: a Q methodological study. BMJ Support Palliat Care. 2017; bmjspcare-2015-000852. doi: 10.1136/bmjspcare-2015-000852.2017.
6. Neilson SJ, Gibson F, Greenfield SM. Pediatric Oncology Palliative Care: Experiences of General Practitioners and Bereaved Parents. J Palliat Care Med. 2015;5(2):214.
7. van der Geest IM, Bindels P, Pluijm S, et al. Palliative care in children with cancer: implications for general practice. Br J Gen Pract. 2016;66(653):599-600.
8. Jarvis SW, Parslow R, Hewitt CE, et al. GPs' role in caring for children and young people with life limiting conditions – a retrospective cohort study. Br J Gen Pract. 2020;70(693): e221-e229. DOI: https://doi.org/10.3399/bjgp20X7082332020:1-9.
9. Mitchell S, Slowther A-M, Coad J, Dale J. The journey through care: study protocol for a longitudinal qualitative interview study to investigate the healthcare experiences and preferences of children and young people with life-limiting and life-threatening conditions and their families in the West Midlands, UK. BMJ Open. 2018;8(1).
10. Murray SA, Kendall M, Carduff E, et al. Use of serial qualitative interviews to understand patients' evolving experiences and needs. Br Med J. 2009;339
11. Braun V, Clarke V. Using thematic analysis in psychology. Qualitative Research in Psychology. 2006;3(2):77-101.
12. Braun V, Clarke V. Successful Qualitative Research. London: SAGE; 2013.
13. Burnard P, Gill P, Stewart K, Treasure E, Chadwick B. Analysing and presenting qualitative data. Br Dent J. 2008;204(8):429-32.
14. van der Geest IM, Bindels PJ, Pluijm SM, et al. Home-Based Palliative Care for Children With Incurable Cancer: Long-term Perspectives of and Impact on General Practitioners. J Pain Sympt Manage. 2017;53(3):578-87.
15. van der Geest IM, Darlington AS, Streng IC, et al. Parents' experiences of pediatric palliative care and the impact on long-term parental grief. J Pain Symptom Manage. 2014;47(6):1043-53.
16. Brown M, Higgins A, MacArthur J. Transition from child to adult health services: A qualitative study of the views and experiences of families of young adults with intellectual disabilities. J Clin Nurs. 2020;29(1-2):195-207.
17. NHS Digital. General and Personal Medical Services, England As at 31 March 2017, Experimental statistics https://digital.nhs.uk/data-and-information/publications/statistical/general-practice-workforce-archive/as-at-31-march-2017-experimental-statistics2018

For peer review only - http://bmjopen.bmj.com/site/about/guidelines.xhtml

Table 1: Summary of study population.

Family	Participants and identifier	Child's age at recruitment	Type of condition	Male or Female	GP practice area	Palliative care service involved	Total number of interviews	Interview details
1	Child (C001)<br>Mother (M001)<br>Father (F001)	5	Cancer	M	Urban	No	4	1: Mother<br>2: Mother and child individual interviews<br>3: Family group
2	Child (C002)<br>Mother (M002)<br>Brother (B002)	17	Congenital	F	Inner-city	Yes	3	3 x family group interviews all including child
3	Mother (M003)<br>Father (F003)	8	Congenital	F	Inner-city	Yes	3	1: Mother<br>2: Both parents<br>3: Mother
4	Father (F004)	8	Congenital	F	Inner-city	Yes	1	1: Father
5	Child (C005)<br>Mother (M005)	6	Congenital	M	Urban	No	3	3 x family group interviews all including child
6	Mother (M006)	18	Congenital	M	Inner-city	No	3	3 x interviews with mother
7	Child (C007)<br>Mother (M007)<br>Father (F007)	7	Cancer	M	Urban	No	5	1: Family group<br>2: Parents and child individually<br>3: Mother and child individually
8	Child (C008)<br>Mother (M008)<br>Brother (B008)	5	Congenital	M	Inner-city	No	4	1: Family group<br>2: Mother and child

For peer review only - http://bmjopen.bmj.com/site/about/guidelines.xhtml

								individual interviews<br>3: Family group
9	Child (C009)<br>Mother (M009)<br>Father (F009)	11	Cancer	F	Inner-city	No	3	3 x family group interviews all including child
10	Mother (M010)	5	Congenital	M	Urban	Yes	1	1: Mother (child present but too unwell to contribute)
11	Child (C011)<br>Mother (M011)<br>Step-father (F011)	17	Congenital	F	Urban	No	4	1: Family group<br>2: Family group<br>3: Family and child individual interviews
12	Child (C012)<br>Mother (M012)	14	Cancer	M	Urban	No	1	1: Family group
13	Child (C013)<br>Mother (M013)	14	Cancer	M	Urban	Yes	3	1: Mother<br>2: Mother and child interviews
14	Child (C014)<br>Mother (M014)	10	Congenital	M	Rural	Yes	3	3 x Family group interviews (including child)

For peer review only

For peer review only - http://bmjopen.bmj.com/site/about/guidelines.xhtml

COREQ (COnsolidated criteria for REporting Qualitative research) Checklist

A checklist of items that should be included in reports of qualitative research. You must report the page number in your manuscript where you consider each of the items listed in this checklist. If you have not included this information, either revise your manuscript accordingly before submitting or note N/A.

Topic	Item No.	Guide Questions/Description	Reported on Page No.
**Domain 1: Research team and reflexivity**			
Personal characteristics			
Interviewer/facilitator	1	Which author/s conducted the interview or focus group?	
Credentials	2	What were the researcher's credentials? E.g. PhD, MD	
Occupation	3	What was their occupation at the time of the study?	
Gender	4	Was the researcher male or female?	
Experience and training	5	What experience or training did the researcher have?	
Relationship with participants			
Relationship established	6	Was a relationship established prior to study commencement?	
Participant knowledge of the interviewer	7	What did the participants know about the researcher? e.g. personal goals, reasons for doing the research	
Interviewer characteristics	8	What characteristics were reported about the inter viewer/facilitator? e.g. Bias, assumptions, reasons and interests in the research topic	
**Domain 2: Study design**			
Theoretical framework			
Methodological orientation and Theory	9	What methodological orientation was stated to underpin the study? e.g. grounded theory, discourse analysis, ethnography, phenomenology, content analysis	
Participant selection			
Sampling	10	How were participants selected? e.g. purposive, convenience, consecutive, snowball	
Method of approach	11	How were participants approached? e.g. face-to-face, telephone, mail, email	
Sample size	12	How many participants were in the study?	
Non-participation	13	How many people refused to participate or dropped out? Reasons?	
Setting			
Setting of data collection	14	Where was the data collected? e.g. home, clinic, workplace	
Presence of non-participants	15	Was anyone else present besides the participants and researchers?	
Description of sample	16	What are the important characteristics of the sample? e.g. demographic data, date	
Data collection			
Interview guide	17	Were questions, prompts, guides provided by the authors? Was it pilot tested?	
Repeat interviews	18	Were repeat inter views carried out? If yes, how many?	
Audio/visual recording	19	Did the research use audio or visual recording to collect the data?	
Field notes	20	Were field notes made during and/or after the inter view or focus group?	
Duration	21	What was the duration of the inter views or focus group?	
Data saturation	22	Was data saturation discussed?	
Transcripts returned	23	Were transcripts returned to participants for comment and/or	

For peer review only

Topic	Item No.	Guide Questions/Description	Reported on Page No.
		correction?	
**Domain 3: analysis and findings**			
Data analysis			
Number of data coders	24	How many data coders coded the data?	
Description of the coding tree	25	Did authors provide a description of the coding tree?	
Derivation of themes	26	Were themes identified in advance or derived from the data?	
Software	27	What software, if applicable, was used to manage the data?	
Participant checking	28	Did participants provide feedback on the findings?	
Reporting			
Quotations presented	29	Were participant quotations presented to illustrate the themes/findings? Was each quotation identified? e.g. participant number	
Data and findings consistent	30	Was there consistency between the data presented and the findings?	
Clarity of major themes	31	Were major themes clearly presented in the findings?	
Clarity of minor themes	32	Is there a description of diverse cases or discussion of minor themes?	

Developed from: Tong A, Sainsbury P, Craig J. Consolidated criteria for reporting qualitative research (COREQ): a 32-item checklist for interviews and focus groups. *International Journal for Quality in Health Care*. 2007. Volume 19, Number 6: pp. 349 – 357

Once you have completed this checklist, please save a copy and upload it as part of your submission. DO NOT include this checklist as part of the main manuscript document. It must be uploaded as a separate file.

For peer review only

BMJ Open

Experiences of General Practice of children with complex and palliative care needs and their families: a qualitative study

Journal:	*BMJ Open*
Manuscript ID	bmjopen-2020-041476.R2
Article Type:	Original research
Date Submitted by the Author:	26-Nov-2020
Complete List of Authors:	Mitchell, Sarah; The University of Sheffield; University of Warwick Warwick Medical School, Academic Unit of Primary Care<br>Harding, Stephanie; University of Warwick<br>Samani, Mohini; NIHR CRN West Midlands Young Persons Steering Group<br>Slowther, Anne-Marie; University of Warwick, Warwick Medical School<br>Coad, Jane; University of Nottingham Faculty of Medicine and Health Sciences<br>Dale, Jeremy; University of Warwick, Warwick Medical School
**Primary Subject Heading**:	General practice / Family practice
Secondary Subject Heading:	Palliative care, Paediatrics
Keywords:	Community child health < PAEDIATRICS, Paediatric palliative care < PAEDIATRICS, PRIMARY CARE, PAEDIATRICS

SCHOLARONE™
Manuscripts

I, the Submitting Author has the right to grant and does grant on behalf of all authors of the Work (as defined in the below author licence), an exclusive licence and/or a non-exclusive licence for contributions from authors who are: i) UK Crown employees; ii) where BMJ has agreed a CC-BY licence shall apply, and/or iii) in accordance with the terms applicable for US Federal Government officers or employees acting as part of their official duties; on a worldwide, perpetual, irrevocable, royalty-free basis to BMJ Publishing Group Ltd ("BMJ") its licensees and where the relevant Journal is co-owned by BMJ to the co-owners of the Journal, to publish the Work in this journal and any other BMJ products and to exploit all rights, as set out in our licence.

The Submitting Author accepts and understands that any supply made under these terms is made by BMJ to the Submitting Author unless you are acting as an employee on behalf of your employer or a postgraduate student of an affiliated institution which is paying any applicable article publishing charge ("APC") for Open Access articles. Where the Submitting Author wishes to make the Work available on an Open Access basis (and intends to pay the relevant APC), the terms of reuse of such Open Access shall be governed by a Creative Commons licence – details of these licences and which Creative Commons licence will apply to this Work are set out in our licence referred to above.

Other than as permitted in any relevant BMJ Author's Self Archiving Policies, I confirm this Work has not been accepted for publication elsewhere, is not being considered for publication elsewhere and does not duplicate material already published. I confirm all authors consent to publication of this Work and authorise the granting of this licence.

For peer review only

Experiences of General Practice of children with complex and palliative care needs and their families: a qualitative study

Mitchell S, Harding S, Samani M, Slowther A-M, Coad J, Dale J.

Authors:

Dr Sarah Mitchell, MBChB FRCGP MSc PhD[1,2],
Miss Stephanie Harding[2],
Miss Mohini Samani[3],
Prof Anne-Marie Slowther MBChB MRCGP MA DPhil[2],
Prof Jane Coad RGN / RSCN PGDip BA BSc PhD[4],
Prof Jeremy Dale MA MBBS PhD FRCGP[2].

Corresponding Author:

Sarah Mitchell
Clinical Research Fellow, University of Sheffield
S.J.Mitchell@sheffield.ac.uk;
@MacGPSarah

Author affiliations:

1. University of Sheffield, Dept of Oncology and Metabolism, Weston Park Hospital, Whitham Road, Sheffield, S10 2SJ
2. Warwick Medical School, University of Warwick, Gibbet Hill Road, Coventry, CV4 7AL
3. NIHR CRN West Midlands Young Person's Steering Group
4. School of Health Sciences, Queens Medical Centre Campus, University of Nottingham, NG7 2HA

Word counts

Abstract: 234
Article: 3,593 (including quotes) (3,054 not including quotes)
Tables: 1

Authorship statement

SM, JD, A-MS and JC designed the study. SM conducted the interviews. SM and SH analysed the data, with input from MS as a representative of the PPI advisory group. JC, A-MS and JD revised the article critically for clarity and intellectual content. All authors have approved this version for submission.

For peer review only - http://bmjopen.bmj.com/site/about/guidelines.xhtml

Abstract

Objectives

To investigate the views and experiences of general practice of children with life-limiting and life-threatening conditions, and their family members, through secondary analysis of a qualitative serial interview study. Thematic analysis was conducted on all interview data relating to experiences of primary care.

Setting

West Midlands, UK.

Participants

A total of 31 participants (10 children with life-limiting and life-threatening conditions and 21 family members) from 14 families.

Study Design and Setting

Secondary thematic analysis of qualitative interview data from a study carried out in the West Midlands, UK.

Method

41 serial interviews with 31 participants from 14 families: 10 children aged 5-18 years with life-limiting and life-threatening conditions, and 21 of their family members.

Results

Three key themes emerged: (1) poor experiences of general practice cause children and families to feel isolated, (2) children and families value support from general practice, and (3) general practice has the potential to provide important aspects of care. Children and families reported benefits from fostering their relationship with their general practice in order to access important aspects of care, including the assessment and management of acute illness, chronic disease and medication reviews, and holistic support.

Conclusion

Children with life-limiting and life-threatening conditions and their families value the involvement of general practice in the care, alongside their paediatric specialists. Ways of developing and providing such support as part of an integrated system of care need to be developed.

Keywords

Primary health care, General practice, Palliative care, Paediatrics, Child health

For peer review only - http://bmjopen.bmj.com/site/about/guidelines.xhtml

Strengths and Limitations

- Little is known about the role of general practice in the care of children with life-limiting and life-threatening conditions and their families, including aspects of palliative care.
- This study provides insights into the support and care from general practice that children and their family members find most helpful from their perspective, including their strategies for accessing such care.
- The study used longitudinal qualitative interviews, which provided the benefit of developing rapport and gaining in-depth insights into the experiences of children and their families.
- There was diversity amongst the children and families interviewed in terms of their conditions, family circumstances, ethnic background and geographical location (innercity, urban and rural).
- This study is a subanalysis of a wider study that did not specifically focus on experiences of general practice, and as such, the dataset is limited, and there is more work to do to understand the specific views of children.

For peer review only

Introduction

The numbers of children and young people (hereafter referred to as "children") aged 0-19 years living with life-limiting conditions (for which there is no reasonable hope of cure) and life-threatening conditions (those for which curative treatment is feasible but may fail) is rising rapidly (1-3). As medical treatments and technology advance, these children are living with increasingly complex conditions. Their health is often fragile, with sudden and unanticipated deterioration leading to admission to hospital and intensive care. This has a huge impact on family life, and presents a number of challenges for healthcare services, including the management of complex clinical issues and the provision of adequate care and support, including palliative care, as close to home as possible (4).

The involvement of general practitioners (GPs) and primary care teams in the provision of healthcare to children with life-limiting and life-threatening conditions can be variable. These children, like all others, have primary care needs in addition to the specialist needs associated with their underlying conditions. Those with life-limiting and life-threatening conditions may also have associated palliative care needs (2). GPs have a key role in the delivery of "core" palliative care to all patients who need it. For children, this would be alongside community nursing and specialist paediatric palliative care colleagues. However, GPs have expressed significant concerns about this role in palliative care for children, including a lack of time to be involved, lack of specialist knowledge (particularly with rare conditions), and understanding of their role alongside paediatric colleagues (5, 6). However, GPs have the potential to provide many vital aspects of palliative care, including the co-ordination of care from community and specialist teams, provision of prescriptions, holistic support for family members (including during bereavement) and support when children transition from paediatric to adult palliative care services (7). Furthermore, frequent appointments with a GP and continuity of GP care for children with life-limiting and life-threatening conditions is associated with less frequent use of urgent and emergency secondary care services (8).

This study aimed to explore the role of general practice in the care of children with life-limiting and life-threatening conditions and their families, from their perspective, and consider how this might be developed to more effectively support the delivery of care to these children in the future.

Method

This study is a secondary analysis of data relating to primary care collected during a qualitative serial interview study carried out from October 2016 to November 2017 in the West Midlands, UK (9). The overall aim of the study was to examine the delivery of healthcare, including palliative care, for children with life-limiting and life-threatening conditions and their families, using a realist approach

For peer review only - http://bmjopen.bmj.com/site/about/guidelines.xhtml

to provide understanding into how palliative care is delivered most effectively, and when, leading to policy relevant recommendations. Longitudinal interviews provided further benefits, including opportunities for rapport building and observation of changing needs and experiences of healthcare over time (10).

Children and family members were provided with study information leaflets eithereither by their clinical teams, or via leaflets and posters in public areas including waiting rooms of hospital outpatient departments. In order to avoid possible coercion, clinical teams did not actively recruit children and families to the study. ChildrenChildren and family members were recruited after either directly approaching the research team viavia email, text or telephone using the details provided in the study information resources, or after expressing an interest in participation and providing permission to be contacted. Each child and family member participated in up to three interviews over the 13-month period, either individually or as a family group. Qualitative research methods were most appropriate in order to elicit and explore the views and perceptions of children and their families. The interview process, including whether children wished to be interviewed alone or with their family members on each occasion, the timing and intervals between interviews, were individually considered according to the needs of each child and family. The interviews were open and conversational, and advanced communication skills, including responding to cues and the use of silence were employed. This allowed exploration of potentially sensitive issues, including experiences of palliative care as both a broad approach to care, and when the child and family had contact with specialist paediatric palliative care services.

Consent procedures were designed with the aim of obtaining written and / or verbal consent and agreement from every individual for every interview. For children under the age of 16, written consent was obtained from the parent and then verbal or written agreement obtained from the child. An agreement to participate form was completed by the child if they chose to do so, in order to respect their autonomy in so far as was possible.

Recordings of interviews were transcribed verbatim, and anonymised transcripts uploaded into NVivo for data management. All data related to experiences of general practice were coded by SM and formed the dataset for this secondary analysis. Two researchers (SH and SM) independently coded the data using an inductive approach to thematic analysis as described by Braun & Clarke (11, 12). This started with a process of familiarisation, reading and re-reading the transcripts, with reflection and note taking. A descriptive code was applied to every item of data and thet emerging codes and concepts were discussed between the researchers weekly throughout the data analysis process, allowing for the development of the themes and decreasing lone researcher bias (13).

For peer review only - http://bmjopen.bmj.com/site/about/guidelines.xhtml

Patient and public involvement (PPI)

PPI was an integral part of the wider study and informed every stage of the research. A group of PPI representatives was recruited from three existing advisory groups (based at Birmingham Children's Hospital, Acorns Children's Hospice and the NIHR Clinical Research Network in the West Midlands). The PPI representatives were aged between nine and 25 years, and included young people with life-limiting conditions and siblings. The group were consulted at all stages of the research, from the study design and objectives to the format of interviews for children, and dissemination activities. A PPI representative (MS) volunteered to support this sub-analysis, providing feedback on the emerging themes and supporting dissemination.

Findings

Study sample

A total of 14 families were recruited to the study, all registered with different GP practices, urban, inner-city and rural. Ten children (aged 5-18 years) and 21 family members took part in the interviews. Children took part in the interviews either with their family members or alone, according to their preference on the day of the interview. Four children were unable to take part either because they could not communicate verbally, or because they were too unwell. 41 interviews were conducted in total. All of the children received care from a local community children's nursing team, and six received care from a specialist paediatric palliative care team or children's hospice service. The study population is summarised in table 1.

[insert Table 1]

Qualitative findings

The children and their family members described a range of experiences of general practice both positive and negative. They did not tend to associate GPs with providing aspects of palliative care; all of the children and families who received palliative care services considered and conceptualised "palliative care" as distinct and separate from other services. Three overarching themes relating to general practice were identified:

Theme 1: Poor experiences of general practice cause children and families to feel isolated

For many, there was a lack of contact with GPs and practice teams. Several families had negative experiences and chose to avoid the GP surgery completely due to these experiences, tending to contact their specialist teams for help instead. Most described their GPs as being of little or "*no help at all*" (M002). Many family members described a "*fight*" to access the support from obtain their GP practice that they required:

For peer review only - http://bmjopen.bmj.com/site/about/guidelines.xhtml

"I haven't got the power, the brain power, to deal with the doctors' surgery. Literally, it's just like I can't. The doctors' surgery is a no go. I avoid it. It will be my last call, if I have to." (M005)

Negative experiences led to a loss of trust in the GP that family members felt unable to reconcile. In these circumstances, there was a sense of unfortunate loss of an important source of support. In one example, the GP was considered responsible for missing a significant diagnosis (cancer) in the child:

"he didn't realise. You know, when you're talking life and death for your child, you can't make mistakes like that. But it was… and most GPs don't see things like this. It's because… they say it's rare, it doesn't feel rare to us in here [the hospital]" (F007).

Some family members had tried to access support from their GP for their own distress related to their child's condition. They sought a listening ear and validation of their distress, but perceived that the GP had limited time to hear and understand, and would focus on providing a medical solution such as an antidepressant tablet. This led to further frustration:

"[the GP] gives me tablets all the time. What if the palpitations are something else? I avoid them now. They are so busy, over-stretched. I think the empathy has gone" (M013)

Another frustration arose on occasions where GPs declined to prescribe regular medications for the children. Family members, who were experts in the management of their children's conditions, found it difficult to understand the rationale for this:

"Everything is like, oh, I can't prescribe this because it's unlicensed, or it's un-this, and simple things, [drug names for emollients], he wouldn't do it" (M005)

Family members recognised that the complexity of their child's condition, and their extensive specialist care, as potential barriers to GPs becoming more involved, with GPs lacking confidence around certain aspects of care, such as prescribing or treating acute medical problems.

"...an awful battle just to try and get antibiotics for her for a urine infection, it was terrible." (M003)

"As soon as [child]'s [life-limiting condition] problems started they just pulled back from everything. Even her basic [medications] and whatever she needed, it all just stopped." (M002)

Even with minor illness, families were advised to access hospital care, which they did not always agree was appropriate:

"...you're bringing this person up to the [children's hospital] who doesn't really need to be at the [children's hospital] and affecting everybody else because the GP won't come out." (M011)

Theme 2: Children and families value support from general practice

For peer review only - http://bmjopen.bmj.com/site/about/guidelines.xhtml

Some families described positive experiences of general practice, considering their GP to be "*realistically the core of it all*" (M003), even when hospital-based, specialist teams mainly provided their care. Some had known a particular GP for many years, and highly valued this continuity, with the GP "*knowing our family*" (M006). For one family, it was the GP who *"finally realised"* (F004) that they were in need of extra support and arranged an onward referral to community children's nursing and palliative care services. Being able to consider the GP as an important member of their healthcare team, through, for example, their attendance when the child had an acute illness, so that they could avoid a trip to hospital, made a significant difference to families where this was the case:

"They're good at coming out if he has a chest infection, they do come out, yeah. Because at the end of the day I've got a child that's on a sats monitor, and ventilator, and now a humidified circuit, and he's not well" (M014)

Children and their family members all expressed a desire for more support from the GP practice. They wished for a proactive offer of support, with recognition of their holistic care needs, through *"a general check on the normal everyday."(M005),* for example through a phone call. This was considered particularly important when there was a long time between hospital appointments. One child described this time as a time when "*no-one knows what is going on*" and specifically regarded the GP or a member of the practice team as someone who could "*check-in*" with him (C013).

"...that would be perfect if there was a somebody there that every six months just said, right, [name], how is everything going in the home, how is everything with .. all your meds, have you got any problems, is there anything I can do for you, can I phone anyone, do you need anything, just a someone." (M013).

Theme 3: Practical ways through which general practice could provide improved care.

Family members described the aspects of care that they wanted general practice teams to provide, and made efforts to "*consciously foster"* (M006) their relationships with GPs in order to access this care. Opportunities existed in chronic disease reviews, appointments for vaccinations and sick notes:

"I have always been quite proactive in taking my children to the GP not only when they're sick. I would take them [children] along for flu jabs and things like that, just so that they knew what they're like, so that when I take them when they're sick they know the difference" (M006)

Interventions that children and family members felt it would be helpful for general practice teams to offer included blood tests, so that they didn't have to travel long distances to hospital. Where repeat prescriptions were a particular cause of difficulty, members of the wider team, including practice

For peer review only - http://bmjopen.bmj.com/site/about/guidelines.xhtml

pharmacists had been instrumental in improving the situation through review of discharge summaries and updating a regular prescription:

"As soon as they get a letter from the hospital to say her meds changed they have someone ring me and we go through her meds." (M011)

Transition to adult services was a cause of concern for many families. They considered this a time when it would be helpful for support from general practice to "*really kick in*" (M003), particularly because organisation of healthcare services for adults differed significantly to those for children:

"I didn't realise that in adult care, a lot of help is from the GP and the community nurse, but we didn't know that because it's different at the [children's hospital]. , and when we went to the [hospital] this consultant, and neuro, said, they [primary care] have to [prescribe the medicines] but they won't do it" (M002)

The need for a consistent and familiar trusted contact within the surgery for children who may want to attend appointments alone, was highlighted:

"how the hell can I transition my son when the GP has never ever called … I'm trying to explain to [child], you will be old enough to go to the GP on your own. That's immense for a normal kid, but a kid with all [his conditions]… he won't talk about all of his…I want him to, because I think it would be a good idea if there was a particular person in the GP's" (M013)

Discussion

Summary

This study provides valuable insights into the highly variable experiences of general practice of children with life-limiting and life-threatening conditions and their family members. For some, there were some positive examples of general practice involvement in the child's care. Generally, the relationship was experienced as unsatisfactory. This could result in a loss of trust, avoidance, or years of battling to obtain the care and support required. Both children and family members expressed a wish to receive more support from their GP practice, and importantly had developed strategies in order to access the support that they found most helpful.

Strengths & limitations

A strength of this study is that it considered the experiences and perceptions general practice from the perspective of children and their families. Family members tended to talk more about experiences of general practice, which is likely to reflect their key involvement in liaising and negotiating services from GPs. This was a subanalysis of a wider study that did not specifically focus on experiences of general practice, and as such, the dataset is limited. Theviews of children and

For peer review only - http://bmjopen.bmj.com/site/about/guidelines.xhtml

family members specifically in relation to general practice are worthy of future research and future studies with a focus on community healthcare, including primary care, are recommended. recommended.

The study population was relatively small, but it was diverse in terms of the children's condition and age. The participants were registered with 14 different practices, in a broad range of different settings. In-depth serial interviews, through which a relationship with children and their families could develop, led to rich, detailed data elicited around many different aspects of care. PPI was gained at several stages of the research, including the development of themes during the analysis to ensure the research remained representative and sensitive.

There is more research to be done to understand the experiences of younger children, young people at transition, and those with non-verbal communication. Further research into the role and response of primary care beyond general practice, including dentists, opticians and out of hours services for children with life-limiting and life-threatening conditions would also be valuable.

Comparison with existing literature

Care for children with complex and palliative care needs in general practice is an under-researched area of practice. The children and families who participated in this study described the particular aspects of care that would be helpful for primary care to provide, and their experiences of how such care could be delivered. The findings are in keeping with the current, limited evidence base, which has explored the experiences of GPs in palliative care of children with cancer (7, 8, 14), and the perspectives of bereaved parents (15). Continuity of care in general practice, effective communication with hospital-based, specialist care, and bereavement support, are areas described repeatedly as needing improvement. Furthermore, the association between more regular contact with GPs and less frequent use of urgent hospital care warrants further investigation in order to understand the factors that result in this outcome for some children, and the details of their relationships with general practice (6). A particularly important area for consideration is transition from paediatric to adult services. This is described as an "overwhelming process" by some parents, with a sense of loss of paediatric services. Continuity of care and support from the GP practice in this scenario has many potential benefits for both parents and children with complex and palliative care needs but is often overlooked (16).

Implications for clinical practice and research

Recent research shows that the number of children with life-limiting or life-threatening conditions increased in England has risen from 32,975 in 2001/2002, to 86,625 in 2017/2018 (3). This is double the total GP headcount in England, which in March 2017 was 41,891 (33,921 whole time equivalent

For peer review only - http://bmjopen.bmj.com/site/about/guidelines.xhtml

GPs) (17). The role and response of general practice in the healthcare of these children and their families, needs greater consideration.

The findings of this study suggest a need for a more systematic proactive approach, such as through prescribing and chronic disease reviews, to provide continuity of care and to build valuable therapeutic relationships with children with life-limiting and life-threatening conditions and their families. GPs and practice team members have the skills and ability to address the physical, psychological, social and spiritual aspects of care, through these relationships and as part of a wider multi-disciplinary team. These aspects of care may not necessarily be associated with "palliative" care, but they provide an important foundation and are integral to the delivery of palliative care to children, which by the nature of children's conditions, may be needed over a prolonged time period. GPs could play an increasingly important role in the identification of palliative care need and onward referral to specialist services, including communication with specialists in paediatric palliative care and children's hospice services. Having a named GP within the practice who becomes the key point of contact for the family and the specialist team, becomes familiar with the needs of the child and family, and undertakes a regular review in which a care plan is agreed, would be of value.

Developing the skills, knowledge, expertise and confidence levels of GPs and other members of the practice team, perhaps those with a particular interest in paediatrics, or management of complexity, to assess acute illness in children with life-limiting and life-threatening conditions would also be of value. In theory, general practice is well placed to work with these children and families, however general practice is under-resourced and may not currently be in a position to prioritise this care.

At a healthcare system level, the need for collaborative partnership working between primary care clinicians and secondary care colleagues in order to improve the experiences of care of children with life-limiting and life-threatening conditions and their families is important. Interventions such as clear and personalised shared care plans, and shared electronic medical records, would be helpful. National professional bodies, including the Royal College of General Practitioners and the Royal College of Paediatrics and Child Health in the UK, have a key role to play in driving forward innovations and effective interventions to facilitate collaborative care. National and local commissioning strategies require focus on this population of children, and should support the delivery of their care in both hospital and community settings.

Conclusion

The findings from this study suggests that general practice should be playing a more important role in the provision of holistic, family-centred healthcare to children with life-limiting and life-threatening conditions and their family members. Children and families described multiple benefits

For peer review only - http://bmjopen.bmj.com/site/about/guidelines.xhtml

to consciously fostering their relationship with general practice clinicians in order to access important aspects of care, including continuity. There are opportunities to engage with this through chronic disease and medication reviews. Clinicians in general practice could develop their skills and expertise in this area to provide assessment and management of acute illness, and long term holistic support, in close partnership with family members and paediatric specialists.

Additional information

Funding statement

This study was funded by a National Institute for Health Research Doctoral Research Fellowship (DRF-2014-07-065).

Disclaimer

This article presents independent research funded in part by the National Institute for Health Research (NIHR). The views expressed are those of the authors and not necessarily those of the NHS, the NIHR or the Department of Health.

Competing interests

The authors have no competing interests

Acknowledgements

The authors would like to acknowledge all of the children and families who took part in this study, and all of the Patient and Public Involvement representatives from Birmingham Children's Hospital, Acorns Children's Hospice and the NIHR CRN West Midlands Young Person's Steering Group who contributed to all aspects of the study.

Ethical approval

Ethical approval was granted by the UK Health Research Authority on 14th September 2016 (IRAS ID: 196816, REC reference: 16/WM/0272, Sponsor: University of Warwick). The data was anonymised and personal identifiers removed to protect confidentiality, as described in the methods.

Data sharing

Individual deidentified participant data that underlie the results published in this article will be made available from 3 months until 3 years following the article publication to investigators with a methodologically sound proposal and whose proposed use of the data has been approved by an independent review committee identified for this purpose, to achieve the aims in the approved proposal. Proposals can be submitted to the corresponding author.

For peer review only

For peer review only - http://bmjopen.bmj.com/site/about/guidelines.xhtml

References

1. Fraser LK, Miller M, Hain R, et al. Rising national prevalence of life-limiting conditions in children in England. Pediatr. 2012;129(4):e923-9.
2. Together for Short Lives. Children's Palliative Care Definitions. https://www.togetherforshortlives.org.uk/changing-lives/supporting-care-professionals/introduction-childrens-palliative-care/categories-of-life-limiting-conditions/.
3. Fraser LK. Gibson-Smith D, Jarvis S. et al. Make Every Child Count. Estimating current and future prevalence of children and young people with life-limiting conditions in the United Kingdom. York: University of York; 2020.
4. Collins A, Hennessy-Anderson N, Hosking S, et al. Lived experiences of parents caring for a child with a life-limiting condition in Australia: A qualitative study. Palliat Med. 2016;30(10):950-9.
5. Neilson S, Gibson F, Jeffares S, Greenfield SM. GPs and paediatric oncology palliative care: a Q methodological study. BMJ Support Palliat Care. 2017; bmjspcare-2015-000852. doi: 10.1136/bmjspcare-2015-000852.2017.
6. Neilson SJ, Gibson F, Greenfield SM. Pediatric Oncology Palliative Care: Experiences of General Practitioners and Bereaved Parents. J Palliat Care Med. 2015;5(2):214.
7. van der Geest IM, Bindels P, Pluijm S, et al. Palliative care in children with cancer: implications for general practice. Br J Gen Pract. 2016;66(653):599-600.
8. Jarvis SW, Parslow R, Hewitt CE, et al. GPs' role in caring for children and young people with life limiting conditions – a retrospective cohort study. Br J Gen Pract. 2020;70(693): e221-e229. DOI: https://doi.org/10.3399/bjgp20X7082332020:1-9.
9. Mitchell S, Slowther A-M, Coad J, Dale J. The journey through care: study protocol for a longitudinal qualitative interview study to investigate the healthcare experiences and preferences of children and young people with life-limiting and life-threatening conditions and their families in the West Midlands, UK. BMJ Open. 2018;8(1).
10. Murray SA, Kendall M, Carduff E, et al. Use of serial qualitative interviews to understand patients' evolving experiences and needs. Br Med J. 2009;339
11. Braun V, Clarke V. Using thematic analysis in psychology. Qualitative Research in Psychology. 2006;3(2):77-101.
12. Braun V, Clarke V. Successful Qualitative Research. London: SAGE; 2013.
13. Burnard P, Gill P, Stewart K, Treasure E, Chadwick B. Analysing and presenting qualitative data. Br Dent J. 2008;204(8):429-32.
14. van der Geest IM, Bindels PJ, Pluijm SM, et al. Home-Based Palliative Care for Children With Incurable Cancer: Long-term Perspectives of and Impact on General Practitioners. J Pain Sympt Manage. 2017;53(3):578-87.
15. van der Geest IM, Darlington AS, Streng IC, et al. Parents' experiences of pediatric palliative care and the impact on long-term parental grief. J Pain Symptom Manage. 2014;47(6):1043-53.
16. Brown M, Higgins A, MacArthur J. Transition from child to adult health services: A qualitative study of the views and experiences of families of young adults with intellectual disabilities. J Clin Nurs. 2020;29(1-2):195-207.
17. NHS Digital. General and Personal Medical Services, England As at 31 March 2017, Experimental statistics https://digital.nhs.uk/data-and-information/publications/statistical/general-practice-workforce-archive/as-at-31-march-2017-experimental-statistics2018

For peer review only - http://bmjopen.bmj.com/site/about/guidelines.xhtml

Table 1: Summary of study population.

Family	Participants and identifier	Child's age at recruit ment	Type of condition	Male or Female	GP practice area	Palliative care service involved	Total number of interviews	Interview details
1	Child (C001)<br>Mother (M001)<br>Father (F001)	5	Cancer	M	Urban	No	4	1: Mother<br>2: Mother and child individual interviews<br>3: Family group
2	Child (C002)<br>Mother (M002)<br>Brother (B002)	17	Congenital	F	Inner-city	Yes	3	3 x family group interviews all including child
3	Mother (M003)<br>Father (F003)	8	Congenital	F	Inner-city	Yes	3	1: Mother<br>2: Both parents<br>3: Mother
4	Father (F004)	8	Congenital	F	Inner-city	Yes	1	1: Father
5	Child (C005)<br>Mother (M005)	6	Congenital	M	Urban	No	3	3 x family group interviews all including child
6	Mother (M006)	18	Congenital	M	Inner-city	No	3	3 x interviews with mother
7	Child (C007)<br>Mother (M007)<br>Father (F007)	7	Cancer	M	Urban	No	5	1: Family group<br>2: Parents and child individually<br>3: Mother and child individually
8	Child (C008)<br>Mother (M008)<br>Brother (B008)	5	Congenital	M	Inner-city	No	4	1: Family group<br>2: Mother and child

For peer review only

For peer review only - http://bmjopen.bmj.com/site/about/guidelines.xhtml

								individual interviews<br>3: Family group
9	Child (C009)<br>Mother (M009)<br>Father (F009)	11	Cancer	F	Inner-city	No	3	3 x family group interviews all including child
10	Mother (M010)	5	Congenital	M	Urban	Yes	1	1: Mother (child present but too unwell to contribute)
11	Child (C011)<br>Mother (M011)<br>Step-father (F011)	17	Congenital	F	Urban	No	4	1: Family group<br>2: Family group<br>3: Family and child individual interviews
12	Child (C012)<br>Mother (M012)	14	Cancer	M	Urban	No	1	1: Family group
13	Child (C013)<br>Mother (M013)	14	Cancer	M	Urban	Yes	3	1: Mother<br>2: Mother and child interviews
14	Child (C014)<br>Mother (M014)	10	Congenital	M	Rural	Yes	3	3 x Family group interviews (including child)

For peer review only

COREQ (COnsolidated criteria for REporting Qualitative research) Checklist

A checklist of items that should be included in reports of qualitative research. You must report the page number in your manuscript where you consider each of the items listed in this checklist. If you have not included this information, either revise your manuscript accordingly before submitting or note N/A.

Topic	Item No.	Guide Questions/Description	Reported on Page No.
**Domain 1: Research team and reflexivity**			
Personal characteristics			
Interviewer/facilitator	1	Which author/s conducted the interview or focus group?	
Credentials	2	What were the researcher's credentials? E.g. PhD, MD	
Occupation	3	What was their occupation at the time of the study?	
Gender	4	Was the researcher male or female?	
Experience and training	5	What experience or training did the researcher have?	
Relationship with participants			
Relationship established	6	Was a relationship established prior to study commencement?	
Participant knowledge of the interviewer	7	What did the participants know about the researcher? e.g. personal goals, reasons for doing the research	
Interviewer characteristics	8	What characteristics were reported about the inter viewer/facilitator? e.g. Bias, assumptions, reasons and interests in the research topic	
**Domain 2: Study design**			
Theoretical framework			
Methodological orientation and Theory	9	What methodological orientation was stated to underpin the study? e.g. grounded theory, discourse analysis, ethnography, phenomenology, content analysis	
Participant selection			
Sampling	10	How were participants selected? e.g. purposive, convenience, consecutive, snowball	
Method of approach	11	How were participants approached? e.g. face-to-face, telephone, mail, email	
Sample size	12	How many participants were in the study?	
Non-participation	13	How many people refused to participate or dropped out? Reasons?	
Setting			
Setting of data collection	14	Where was the data collected? e.g. home, clinic, workplace	
Presence of non-participants	15	Was anyone else present besides the participants and researchers?	
Description of sample	16	What are the important characteristics of the sample? e.g. demographic data, date	
Data collection			
Interview guide	17	Were questions, prompts, guides provided by the authors? Was it pilot tested?	
Repeat interviews	18	Were repeat inter views carried out? If yes, how many?	
Audio/visual recording	19	Did the research use audio or visual recording to collect the data?	
Field notes	20	Were field notes made during and/or after the inter view or focus group?	
Duration	21	What was the duration of the inter views or focus group?	
Data saturation	22	Was data saturation discussed?	
Transcripts returned	23	Were transcripts returned to participants for comment and/or	

For peer review only

Topic	Item No.	Guide Questions/Description	Reported on Page No.
		correction?	
**Domain 3: analysis and findings**			
Data analysis			
Number of data coders	24	How many data coders coded the data?	
Description of the coding tree	25	Did authors provide a description of the coding tree?	
Derivation of themes	26	Were themes identified in advance or derived from the data?	
Software	27	What software, if applicable, was used to manage the data?	
Participant checking	28	Did participants provide feedback on the findings?	
Reporting			
Quotations presented	29	Were participant quotations presented to illustrate the themes/findings? Was each quotation identified? e.g. participant number	
Data and findings consistent	30	Was there consistency between the data presented and the findings?	
Clarity of major themes	31	Were major themes clearly presented in the findings?	
Clarity of minor themes	32	Is there a description of diverse cases or discussion of minor themes?	

Developed from: Tong A, Sainsbury P, Craig J. Consolidated criteria for reporting qualitative research (COREQ): a 32-item checklist for interviews and focus groups. *International Journal for Quality in Health Care*. 2007. Volume 19, Number 6: pp. 349 – 357

Once you have completed this checklist, please save a copy and upload it as part of your submission. DO NOT include this checklist as part of the main manuscript document. It must be uploaded as a separate file.

For peer review only
